# Supplementary figures and images for: A Comparison of Tools for Copy-Number Variation Detection in Germline Whole Exome and Whole Genome Sequencing Data
Source: Cancers (Basel). 2021 Dec 14;13(24):6283. doi: 10.3390/cancers13246283 (PMC8699073; doi:10.3390/cancers13246283)

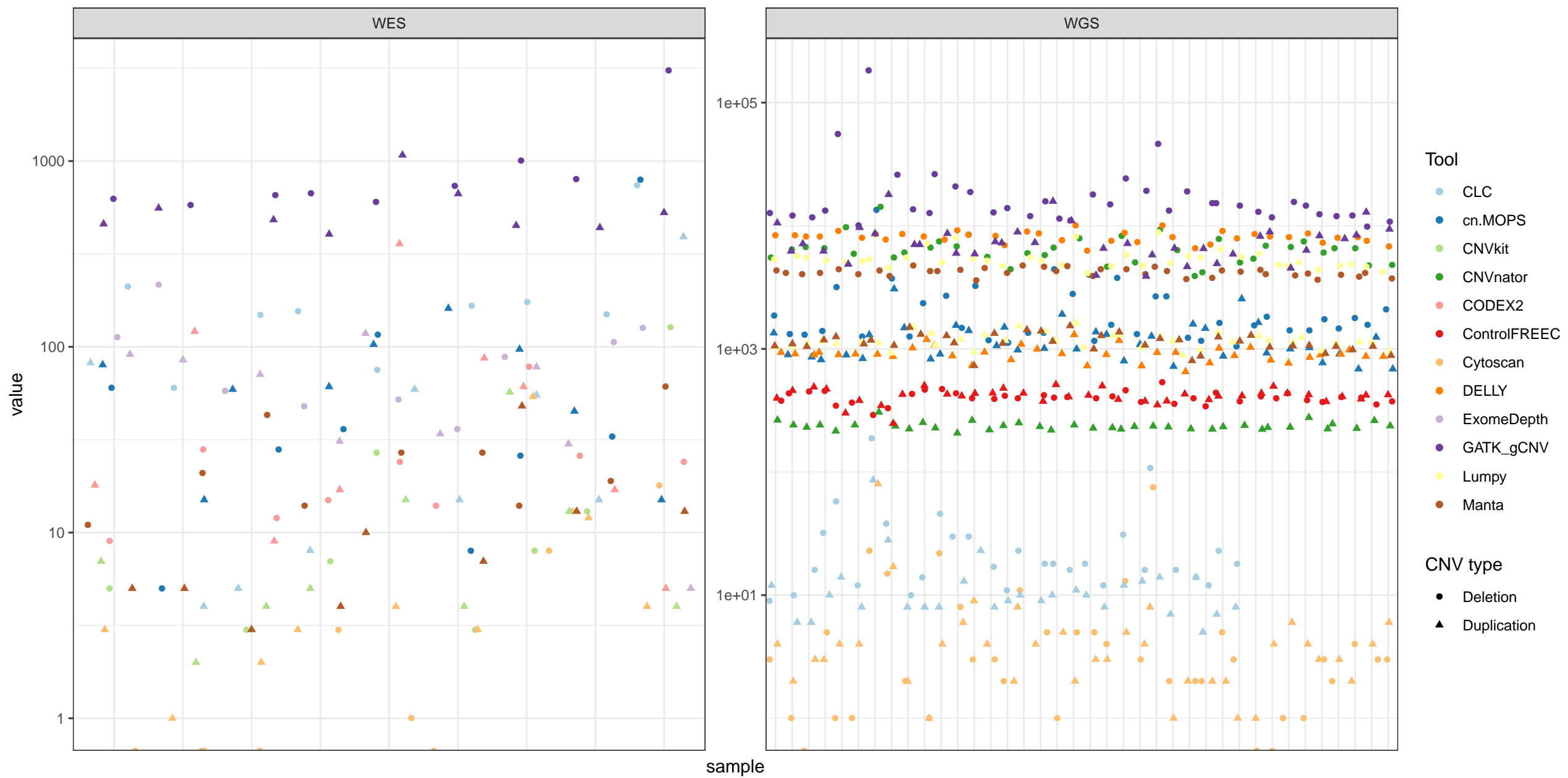

Supplement: Supplementary file 1 [file cancers-13-06283-s001.zip › Supplementary Materials/Figure S1.pdf]

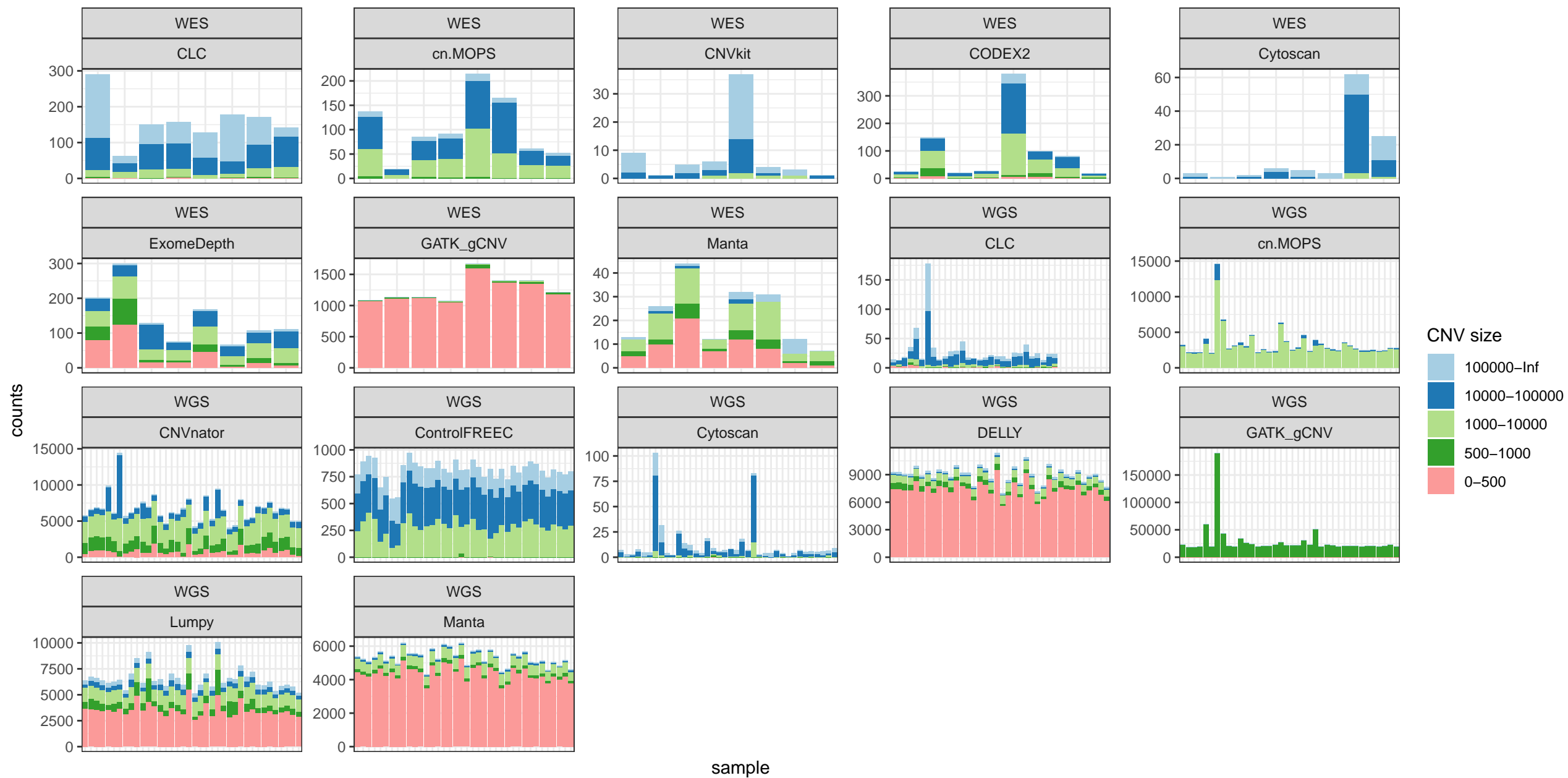

Supplement: Supplementary file 1 [file cancers-13-06283-s001.zip › Supplementary Materials/Figure S2.pdf]
